# *Pandoraea* sp. Strain E26: Discovery of Its Quorum-Sensing Properties via Whole-Genome Sequence Analysis

**DOI:** 10.1128/genomeA.00565-15

**Published:** 2015-05-28

**Authors:** Kok-Gan Chan, Wai-Fong Yin, Kok Keng Tee, Chien-Yi Chang, Kumutha Priya

**Affiliations:** a Division of Genetics and Molecular Biology, Institute of Biological Sciences, Faculty of Science, University of Malaya, Kuala Lumpur, Malaysia; b Centre of Excellence for Research in AIDS (CERiA), Department of Medicine, Faculty of Medicine, University of Malaya, Kuala Lumpur, Malaysia; c Interdisciplinary Computing and Complex BioSystems (ICOS) Research Group, School of Computing Science, Claremont Tower, Newcastle University, Newcastle upon Tyne, United Kingdom; d Centre for Bacterial Cell Biology, Medical School, Newcastle University, Newcastle upon Tyne, United Kingdom

## Abstract

We report the draft genome sequence of *Pandoraea* sp. strain E26 isolated from a former landfill site, sequenced by the Illumina MiSeq platform. This genome sequence will be useful to further understand the quorum-sensing system of this isolate.

## GENOME ANNOUNCEMENT

*Pandoraea* is a member of the *Burkholderiaceae* family and is a nonsporulating, straight-rod-shaped, Gram-negative betaproteobacterium. It is motile by means of a single polar flagellum (1). Several *Pandoraea* spp. have been found to cause detrimental and irreversible damage to the respiratory system, especially in cystic fibrosis (CF) patients. Multiple case reports published on *Pandoraea* spp. identify them as some of the pathogens responsible for chronic lung infections alongside the infamous *Pseudomonas aeruginosa* (2–5). The severity of infection by *Pandoraea* spp. is compounded by their misidentification as *Ralstonia* sp. and *Burkholderia cepacia* by clinicians (6). This further endangers the life of the patient, as misidentification causes the prescription of the wrong treatment. The availability of the whole-genome sequences of *Pandoraea* sp. strain E26 will help researchers understand the complexity and pathogenicity of this species as well as prevent further misidentification.

*Pandoraea* sp. strain E26 was isolated from the soil surface of a former landfill site in Ayer Hitam, Puchong, Malaysia using a KGM growth medium (7). Preliminary studies using quorum-sensing biosensors, *Chromobacterium violaceum* CVO26 and *Escherichia coli*(pSB401), showed that this isolate possesses quorum-sensing activities (8, 9). However, no quorum-quenching activity was observed. High-resolution tandem mass spectrometry (LC-MS/MS) detected *N-*octanoyl homoserine lactone (C8-HSL) from the spent supernatant of this isolate.

Genomic DNA of *Pandoraea* sp. strain E26 was extracted and purified using a QIAamp DNA minikit (Qiagen, Germany) per the manufacturer’s protocol. The quality of the genomic DNA was assessed using a NanoDrop spectrophotometer (Thermo Scientific) and Qubit 2.0 fluorometer (Life Technologies). Purified and normalized genomic DNA of the isolate was subjected to whole-genome-shotgun sequencing on an Illumina MiSeq (Illumina, Inc., CA) platform. The raw sequence data were trimmed and subsequently assembled using CLC Genomics Workbench version 7.5 (CLC Bio, Denmark). Sequence reads of low quality with a cutoff value of <Q30 and ambiguous nucleotides were trimmed prior to assembly. Genome annotation was performed using Prokka (10).

The whole-genome sequencing generated 3,204,829 paired-end reads with an average read length of 130.3 bp. The assembly of the genome yielded 96 contigs with an average contig size of 57.1 kb, with the largest contig size of 773.2 kb. The final draft genome of *Pandoraea* sp. strain E26 contained 5,476,952 bases with approximately 62× coverage and a G+C content of 64.7%. Gene annotation using Prokka resulted in 4,834 coding sequences (CDSs). A total of 57 tRNAs were predicted as well as one copy each of a 5S rRNA gene, 23S rRNA gene, and 16S rRNA gene. Based on the gene annotation results, an autoinducer synthase gene was detected in contig 26 of the draft genome of *Pandoraea* sp. E26.

Members of *Pandoraea* show various activities such as oxalate-degradation (11), pathogenesis (12), and quorum sensing (13). *Pandoraea pnomenusa* (12) could be the source of problems in cystic fibrosis patients. The availability of the whole-genome sequences of *Pandoraea* spp. could be useful to provide a better understanding of this lesser known bacterium.

### Nucleotide sequence accession numbers.

This whole-genome shotgun project has been deposited at DDBJ/EMBL/GenBank under the accession no. AYXJ00000000. This version described in this paper is the version AYXJ01000000.
